# Microbial Interactions between Amylolytic and Non-Amylolytic Lactic Acid Bacteria Strains Isolated during the Fermentation of Pozol

**DOI:** 10.3390/foods10112607

**Published:** 2021-10-28

**Authors:** Sandra Bolaños-Núñez, Jorge A. Santiago-Urbina, Jean-Pierre Guyot, Gloria Díaz-Ruiz, Carmen Wacher

**Affiliations:** 1 Departamento de Alimentos y Biotecnología, Facultad de Química, Universidad Nacional Autónoma de México, Ciudad de México 04510, Mexico; zandybn@gmail.com (S.B.-N.); gloriadr@unam.mx (G.D.-R.); 2 División de Dirección de Carrera de Agricultura Sustentable y Protegida, Universidad Tecnológica de los Valles Centrales de Oaxaca, Zimatlán, Oaxaca 71270, Mexico; jorgesantiago.urbina@gmail.com; 3 Institut de Recherche pour le Développement, UMR 204 Nutripass, 34394 Montpellier, France; nutripass@ird.fr

**Keywords:** pozol, lactic acid bacteria, fermentation, starch, microbial dynamics

## Abstract

Pozol is a Mexican beverage prepared from fermented nixtamalized maize dough. To contribute to understanding its complex microbial ecology, the effect of inoculating on MRS-starch pure and mixed cultures of amylolytic *Sii*-25124 and non-amylolytic *W. confusa* 17, isolated from pozol, were studied on their interactions and fermentation parameters. These were compared with *L. plantarum* A6, an amylolytic strain isolated from cassava. Microbial growth, kinetic parameters, amylolytic activity, lactic acid production, and hydrolysis products from starch fermentation were measured. The population dynamics were followed by qPCR. *L. plantarum* A6 showed higher enzymatic activity, lactic acid, biomass production, and kinetic parameters than pozol LAB in pure cultures. Mixed culture of each pozol LAB with *L. plantarum* A6 showed a significant decrease in amylolytic activity, lactic acid yield, specific growth rate, and specific rate of amylase production. The interaction between *Sii*-25124 and *W. confusa* 17 increased the global maximum specific growth rate (µ), the lactic acid yield from starch (Y_lac/s),_ lactic acid yield from biomass (Y_lac/x_), and specific rate of lactic acid production (q_lac_) by 15, 30, 30, and 40%, respectively, compared with the pure culture of *Sii*-25124. Interactions between the two strains are essential for this fermentation.

## 1. Introduction

Fermented cereals play an essential role in human nutrition in all parts of the world [1,2]. In Southeastern México, pozol, a traditional refreshing non-alcoholic beverage based on nixtamalized maize, is consumed [3]. It is a popular beverage that is part of the diet, especially in rural households and village communities. The process of production involves cooking maize in an alkaline solution containing 1% lime (CaO). These cooked corn grains (named nixtamal) are then washed with water and milled to make a dough called masa or nixtamal dough. It is shaped into balls, wrapped in banana plant leaves, and left at ambient temperature, usually 30–35 or even 40 °C, for 3 to 5 h, one week, one month, or more. It is consumed immediately after preparing it or after fermentation [4]. Pozol is suspended in water and consumed. A natural fermentation, without an intentional inoculum, but knowing that most microorganisms are introduced into the dough during the milling process, occurs [5]. Microbial groups, mainly lactic acid bacteria (LAB), enterobacteria, *Bacillus* spp., yeasts, and molds, have been detected [3,6,7,8,9]. This process is dominated by LAB, mainly *Streptococcus* genus [6,8]. The first day of fermentation is characterized by the presence of amylolytic lactic acid bacteria (ALAB), such as *Streptococcus bovis* [8,10], identified now as *Sii*-25124 (*Streptococcus infantarius* ssp. *infantarius*); smaller concentrations of *Lactobacillus fermentum* (new name *Limosilactobacillus fermentum*) [11], *Lactobacillus plantarum* (new name *Lactiplantibacillus plantarum*) [11], *Enterococcus*, *Leuconostoc*, and *Lactococcus* [8,9]. These LAB are essential microorganisms for pozol fermentation, as most of them can use starch [8,10], the most abundant constituent of maize.

LAB contributes to the stability of the product by inhibiting unwanted microorganisms [12]. They also develop characteristics such as texture and impact in the product’s sensory attributes [13,14].

*Sii*-25124 produces two amylases to metabolize starch, a cytoplasmic α-amylase, and an extracellular amylopullulanase [15]. This extracellular enzyme hydrolyzes starch in the dough to produce oligosaccharides that allow the growth of other LAB species. Thus, it has been postulated that ALAB plays a vital role in making starch available for other microorganisms [6], as non-amylolytic LAB. In pozol, *Weissella confusa* is the most frequent non-amylolytic LAB reported [6,7,16]. Strains of these have been characterized by the gene needed to metabolize other carbohydrates different from the starch such as sucrose, hemicellulose, cellulose, and starch residues [16].

*Streptococcus* and *Weissella* have been detected in most of the samples of pozol that have been analyzed [6,8]. Pozol fermentation is a complex process, as it is based on a substrate that contains different kinds of compounds and a varied microbiota, which most likely interact.

This study aimed to determine the presence of bacterial interactions between amylolytic and non-amylolytic LAB. We aim to determine if growth in MRS-glucose or MRS-starch, and amylolytic activity of pure and mixed cultures of predominant bacterial species previously isolated from pozol: *Sii*-25124 (amylolytic), *W. confusa* 17 (non-amylolytic), and *Lactobacillus plantarum* A6 (*new name Lactiplantibacillus plantarum*), a highly amylolytic strain isolated from cassava (as a control), depending on the use of pure and mixed cultures of these bacteria.

## 2. Materials and Methods

### 2.1. Bacterial Strains

*Streptococcus infantarius* ssp. *infantarius* 25124, an amylolytic LAB, and *Weissella confusa* 17, a non-amylolytic LAB isolated from pozol [8,17] were used. In addition, *Lactobacillus plantarum* A6 (new name *Lactiplantibacillus plantarum*), previously isolated during cassava retting [18] kindly provided by Dr. Jean-Pierre Guyot (Institut de Recherche pour le Développement, Montpellier, France), was used as a positive control for amylolytic activity. All strains were maintained in the stock culture collection of the laboratory and kept in 30% glycerol at −70 °C.

### 2.2. Inoculum Preparation

The strains were reactivated by streaking onto de Man Rogosa and Sharpe (MRS) agar (BD Difco, Sparks, MD, USA) and incubated at 30 °C for 48 h. Then, they were cultivated in 10 mL of appropriated culture media. *W. confusa* 17 was inoculated in MRS-glucose broth (MRS-G; BD Difco, Sparks, MD, USA); while *Sii*-25124 and *L. plantarum* A6 were grown in modified-MRS-starch (tryptone (10 g/L; BD-Difco, Sparks, MD, USA), ammonium citrate (2.17 g/L; JT Baker, Phillipsburg, NJ, USA), sodium acetate (5 g/L; JT Baker, Phillipsburg, NJ, USA), magnesium sulphate heptahydrate (0.207 g/L; Sigma, St. Louis, MO, USA), manganese sulphate (0.056 g/L; Sigma, St. Louis, MO, USA), dipotassium phosphate (2.62 g/L; JT Baker, Phillipsburg, NJ, USA), meat extract (10 g/L; BD-Difco, Sparks, MD, USA), yeast extract (5 g/L; BD-Difco, Sparks, MD, USA), and starch (20 g/L; Prolabo-Merck Eurolab, France). All strains were incubated at 30 °C for 24 h. For the inoculum preparation, 10 mL of the previously activated strain were transferred to 100 mL of MRS-S or MRS-G according to the strain and incubated at 30 °C overnight. The cultures were used as starters for the following experiments.

### 2.3. Monoculture Fermentation

Fermentations of MRS-S broth using pure and mixed cultures were carried out in 1 L Erlenmeyer flasks containing 800 mL of culture medium at 30 °C for 24 h, statically. The sterilized medium was inoculated with the pure cultures grown overnight to start at a concentration of 1 × 10^6^ UFC/mL, corresponding to an optical density of 0.1 at OD (600 nm). All experiments were performed in triplicate.

### 2.4. Mixed Culture Fermentations

Three different mixed cultures were performed for the assessment of bacterial interactions. The first mixed culture consisted of *Sii*-25124 and *L. plantarum* A6, the second mix included *Sii*-25124 and *W. confusa* 17, while the third involved *L. plantarum* A6 and *W. confusa* 17. Both strains were inoculated simultaneously to MRS-S broth in a ratio of 1:1 to achieve approximately 1 × 10^6^ cells/mL. Fermentation was performed at 30 °C for 24 h, statically. All experiments were performed in triplicate.

### 2.5. Sample Collection and pH Determination

During the first seven h of fermentation, samples were collected every 30 min. After that, samples were taken every hour until 12 h after the fermentation had started, wherin a final sample was collected at 26 h of fermentation. The pH and biomass determination of each sample were recorded. The pH of the samples was measured in triplicates using a Jenway 3020 glass electrode pH meter standardized with pH buffer solutions.

### 2.6. Biomass Determination

A calibration curve of optical density at 600 nm (OD_600_) and cell dry weight was established for each strain. Biomass concentration was determined by measuring the OD_600_ with Spectronic 21D spectrophotometer (Milton Roy) and related to dry weight measured after two washing and centrifugation cycles, followed by a drying step at 80 °C for 24 h. A calibration curve was previously performed between the optical density and the dry weight.

### 2.7. Determination of α-Amylase Activity

For determination of α-amylase activity, 10 mL of the fermented broth were centrifuged at 10,000 rpm, 4 °C for 15 min. To detect cell-bound amylase activity of *Sii*-25124, cell pellets were recovered, washed, and suspended in 0.1 M phosphate buffer (pH 6.8). Amylase activity was assayed at pH 6.8 and 37 °C by measuring the iodine-complexing ability of starch as described by Agati et al. [19]. To determine the amylase activity of *L. plantarum* A6, 10 mL of fermented broth was centrifuged at 10,000 rpm, 4 °C for 15 min, and the cell-free supernatant was recovered as a crude enzyme extract. Enzymatic activity was tested at pH 5.0 and 65 °C using the starch-iodine extinction method [20]. The lack of amylolytic activity of *W. confusa* 17, was proved on cells and on the supernatant.

### 2.8. HPLC Analysis

Samples were centrifuged at 10,000 rpm for 10 min, and supernatants were filtered through a 0.22 μm Millipore membrane filter (EMD Millipore, Billerica, MA, USA). Lactic acid and starch hydrolysis products were determined with the liquid chromatography system (Perkin Elmer 250, Norwalk, CT, USA), equipped with a refractive index detector (Perkin-Elmer 30, Norwalk, CT, USA). Anion-exclusion aminex HPX-87H column (300 × 7.8 mm; Bio-Rad, Hercules, CA, USA) was used. Sulfuric acid (0.01 N) was used as the mobile phase at a flow rate of 0.6 mL/min and a column temperature of 50 °C. L-Lactate (L-222; Sigma, Saint Louis, MO, USA) was used as standard. Analysis of hydrolysis products from starch fermentation was performed using high-performance liquid chromatography equipped with the refractive index detector and a Prodigy 5 ODS 2 C18 column (250 × 4.6 mm; Phenomenex, Torrance, CA, USA). The products were eluted with water at a flow rate of 0.8 mL/min and a column temperature of 35 °C. Glucose, maltose, maltotriose, maltotetraose, maltopentaose, maltohexaose, and maltoheptaose were identified by comparing their retention time with standards (Sigma, Saint Louis, MO, USA).

### 2.9. Sugars Quantification

Total and reducing sugars were determined using the Dubois et al. [21] and Miller methods [22].

### 2.10. Kinetic Parameters

The maximum specific growth rate, product and growth yields relative to the substrate (Y_lac/s_ and Y_x/s,_ respectively), lactic acid and amylase yields from biomass (Y_lac/x_ and Y_amy/x_, respectively), specific rates of lactic acid or amylase production (q_lac_ and q_amy_, respectively), and substrate consumption (q_s_) were calculated as indicated by Díaz-Ruiz et al. [8].

Significant differences among the different cultures were determined for kinetic parameters by one-way analysis of variance (LSD, α = 0.05). The statistical software Statgraphics Centurion XVI.I.

### 2.11. DNA Extraction

DNA from pure and mixed culture was extracted using the MagMax™ Nucleic Acid isolation kits (Ambion^®^, Thermo Fisher Scientific Baltics UAB, Vilnius, Lithuania). Twenty milliliters of each culture were centrifuged for 15 min at 10,000 rpm at 4 °C. Pellets were washed three times with sterile distilled water, and each sample was resuspended in 1 mL of sterile distilled water. Bacterial suspensions were stored at −21 °C or directly processed according to the manufacturer’s instructions.

### 2.12. Real-Time PCR Primer Design and Assay Conditions

Primers for detecting phenylalanine transfer RNA (tRNAphe) gene sequences of *S.ii* 25124, *L. plantarum* A6, and *W. confusa* 17 were designed by Applied Biosystems.

Real-time PCR assays were performed on an Applied Biosystems Prism 7500 Real-Time PCR System (Applied Biosystems^TM^, Foster City, CA, USA). qPCR was realized using HotStart-IT SYBR Green qPCR Master Mix (Thermo Scientific, Carlsbad, CA, USA). The 25 µL reaction volume consisted of 12.5 μL of 2X HotStart-IT SYBR Green qPCR Master Mix, 0.5 μM of forward and reverse primers, and 100 to 0.01 ng of DNA were mixed. PCR conditions were denaturation at 95 °C for 10 min, followed by 40 cycles of amplification: 15 s at 95 °C, 1 min at 60 °C, and 72 °C for 1 min. Fluorescence was detected at the end of the elongation phase for each cycle.

### 2.13. Calibration of Standards

Serial dilutions from 100 to 0.1 ng/μL of DNA from single strains samples (*Sii*-25124, *L. plantarum* A6, and *W. confusa* 17) were amplified using the primers and the abovementioned conditions. DNA was quantified with a nanodrop spectrophotometer (Agilent 8453). Each reaction was run in triplicate. Amplification efficiency was determined by plotting the threshold cycle for a 10-fold dilution series against the logarithm of the DNA concentration. Efficiency (E) of the qPCR assay was calculated using the equation E = [10^(−1/slope) − 1] × 100, where the slope refers to the slope of the standard curve for the dilutions series used in qPCR assay. DNA concentration of pure LAB was quantified using the mean values of CT obtained in three independent assays.

## 3. Results

### 3.1. Growth of Amylolytic and Non-Amylolytic-LAB in Pure and Mixed Culture

**Figure 1:**
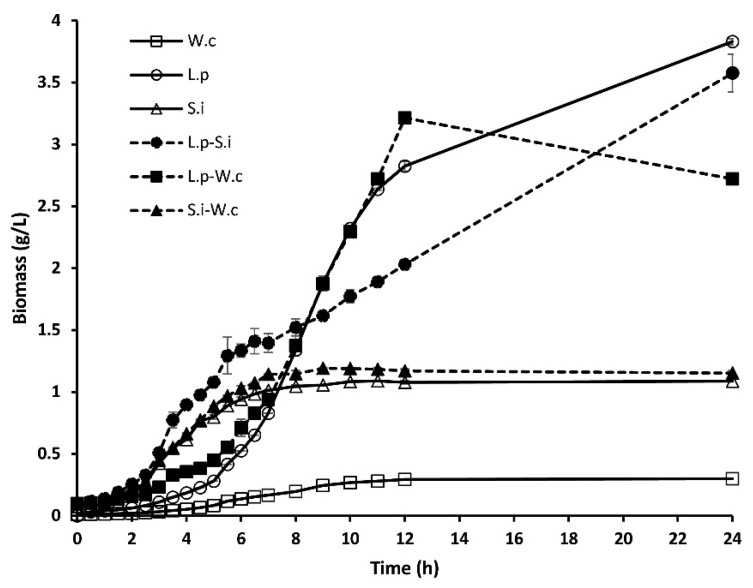
Biomass concentration during fermentation of MRS-starch broth at 30 °C using pure and mixed cultures of *L. plantarum* A6 (new name *Lactiplantibacillus plantarum*), *Sii*-25124, and *W. confusa* A17. Results are means of three independent fermentations. W.c, *W. confusa* 17; L.p, *L. plantarum* A6; S.i, *Sii*-25124. Vertical lines show standard deviations.

The ability of amylolytic and non-amylolytic LAB to grow on MRS-starch broth was evaluated in pure and mixed cultures during the first seven h of fermentation. As pure cultures, *Sii*-25124 presented a higher growth rate than that of *L. plantarum* A6 or *W. confusa* 17 (Figure 1). After this time, these LAB (*Sii*-25124) remained in stationary phase, producing a maximum biomass concentration of 1.08 g/L, while that of *L. plantarum* A6 was 3.8 g/L after 24 h of fermentation. A similar biomass concentration (3.6 g/L) was produced with *L. plantarum* A6 and *Sii*-25124 co-culture. In the early stages of fermentation, this mixed culture showed the highest growth rate than those achieved by other pure or mixed culture fermentation (Figure 1). From 8 to 12 h of the experiment, *L. plantarum* A6 in co-fermentation with *W. confusa* 17 showed higher biomass concentration (3.2 g/L) than the co-culture *L. plantarum* A6-*Sii*-25124, and *Sii*-25124-*W. confusa* 17.

**Figure 2:**
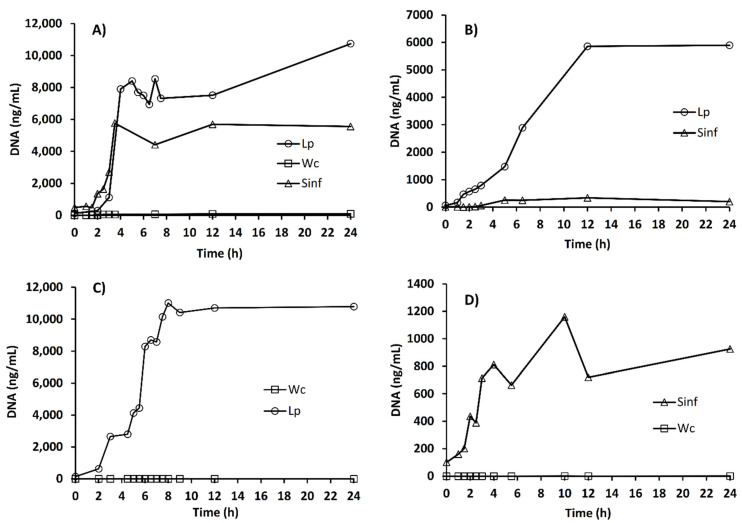
Growth, measured by DNA (Quantitative PCR) of *L. plantarum* A6 (new name *Lactiplantibacillus plantarum*), (Lp), *Sii.* 25,124 (Sinf) and *W. confusa* 17 (Wc) DNA during the fermentation. (**A**) Fermentation performed by pure cultures; (**B**) fermentation performed by *L. plantarum* A6 and *Sii*-25124; (**C**) fermentation performed by *L. plantarum* A6 and *W. confusa* 17; (**D**) fermentation performed by *Sii*-25124 and *W. confusa* 17.

In previous experiments, biomass was measured as the dry weight of either single or mixed cultures. To detect the growth dynamics and effects of each of the inoculated LAB during MRS-starch broth fermentation, real-time PCR assay was also employed, using a specific probe for each strain. Findings were in accordance with the information obtained using biomass determination by dry weight. In pure culture, *L. plantarum* A6 reached the highest DNA concentration (~10,000 ng/mL), followed by *Sii*-25124 (Figure 2A), whereas *W. confusa* 17 did not grow. As shown in Figure 2B, in mixed fermentation with *L. plantarum* A6 and *Sii*-25124, the first species reached ~6000 ng/mL DNA, and the second one only increased to ~300 ng/mL. Thus, they coexisted, but *L. plantarum* A6 dominated the fermentation. However, in the mixed cultures of *W. confusa* 17 with *L. plantarum* A6 or with *Sii*-25124, *W. confusa* 17 was excluded from the fermentation, which *L. plantarum* A6 dominated, that reached ~10,500 ng/mL DNA (Figure 2C), a similar concentration as that obtained as a pure culture. In the mixed culture with *Sii*-25124 and *W. confusa* 17, starch fermentation was dominated by *Sii*-25124 (Figure 2D). However, the DNA concentration was only one sixth (~900 ng/mL) of the concentration reached as a pure culture (~6000 ng/mL, Figure 2A).

### 3.2. Starch Hydrolysis

**Figure 3:**
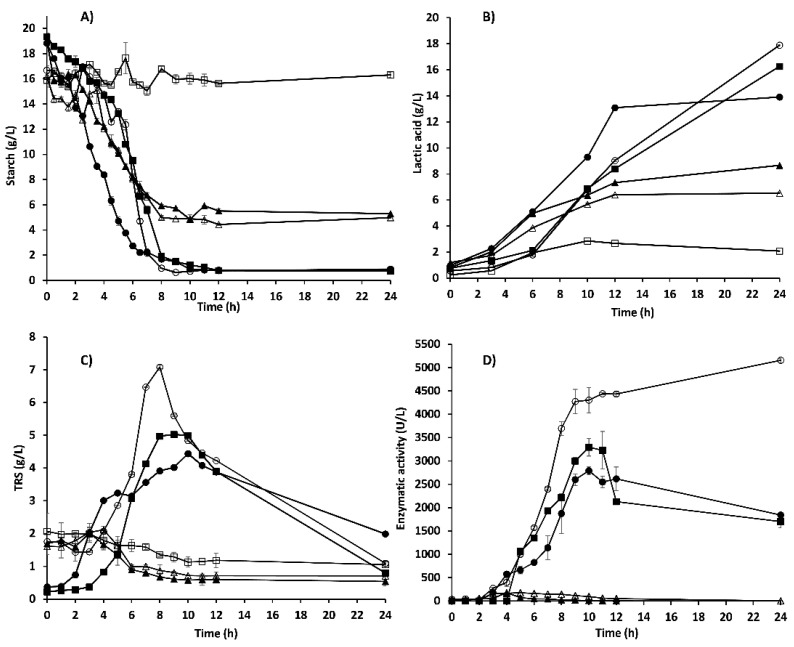
Changes in concentrations of (**A**) starch, (**B**) lactic acid, (**C**) total reducing sugar, and (**D**) enzymatic activity during MRS-starch broth fermentation. *L. plantarum* A6 (new name *Lactiplantibacillus plantarum*), (○); *Sii*-25124 (△); *W. confusa* 17 (□); *L. plantarum* A6-*Sii*-25124 (●); *L. plantarum* A6-*W. confusa* 17 (■); *Sii*-25124-*W. confusa* 17 (▲) at 30 °C. Results are means of three independent fermentations.

As expected, *Weissella confusa* 17 could not hydrolyze starch present in the medium (Figure 3A). However, from 2.5 to six h of fermentation, the mixed culture of *L. plantarum* A6 and *Sii*-25124 presented the fastest starch hydrolysis. Whenever *L. plantarum* A6 was present, the starch was completely hydrolyzed (residual starch below 1 g/L) from 12 h of the assay. On the other hand, *Sii*-25124 in monoculture and co-culture with *W. confusa* 17 could not hydrolyze starch completely (4.8 and 5.29 g/L of residual starch, respectively).

Reducing sugars were produced in concentrations of 4 to 7 g/L (Figure 3C). The maximum sugar concentration was in accordance with the lower starch concentration in the medium from 6 to 12 h of trial. In the case of *W. confusa* 17, *Sii*-25124, and their mixture, reducing sugar concentration did not increase (Figure 3C).

### 3.3. Amylolytic Activity and Starch Hydrolysis Products

**Figure 4:**
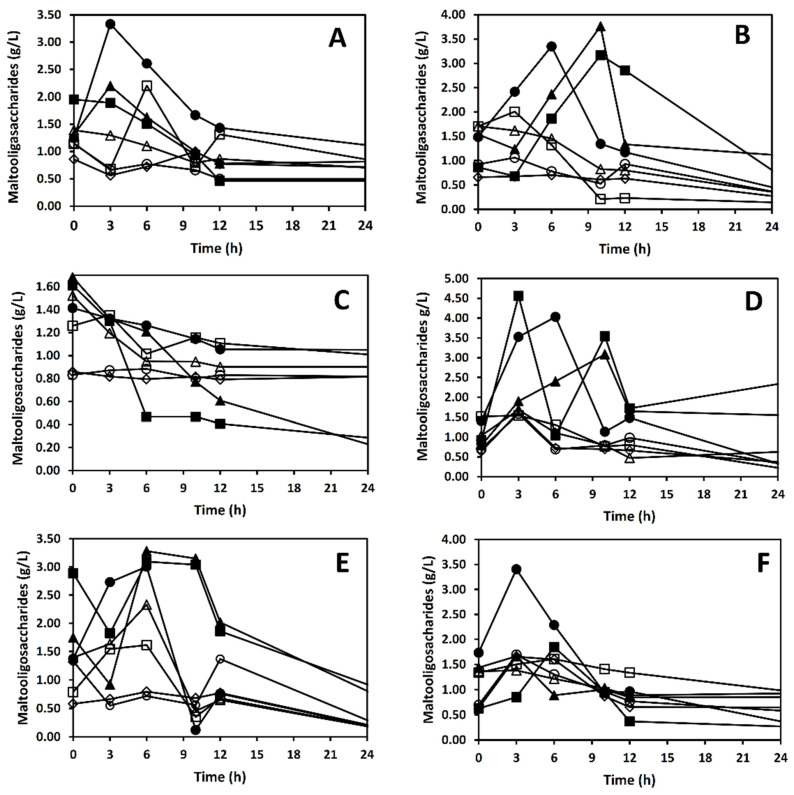
Hydrolysis products from starch fermentation using (**A**) *Sii*-25124; (**B**) *L. plantarum* A6 (new name *Lactiplantibacillus plantarum*); (**C**) *W. confusa* 17; (**D**) *L. plantarum* A6-*Sii*-25124; (**E**) *L. plantarum* A6-*W. confusa* 17; (**F**) *Sii*-25124-*W. confusa* 17. Glucose (▲), maltose (■), maltotriose (●), maltotetraose (△), maltopentaose (□), maltohexaose (○), maltoheptaose (◊).

In monoculture, *Sii*-25124 showed low enzymatic activity during the first two hours of fermentation. After five h, this activity reached 176 U/L (Figure 3D), and then decreased to 1 U/L. Thus, during the first six h of fermentation, an increase in maltopentaose, maltotriose, and glucose concentration (starch hydrolysis products) were detected (Figure 4A). The amylolytic activity of the single and mixed cultures of *L. plantarum* A6 was higher than that of other cultures (Figure 3D). At nine h of fermentation, the amylolytic activity reached a value of 4273 U/L. After this time, the activity increased slowly until 5155 U/L at 24 h (Figure 3D). These results were in accord with the data obtained from the quantification of maltooligosaccharides. It was found that maltose, maltotriose, and glucose concentrations increased during the third and 12 h of fermentation (Figure 4B). In the mixed culture of *Sii*-25124 and *W. confusa* 17, the maximum enzymatic activity (176 U/L) was reached at four h of fermentation. From this point, the amylolytic activity decreased rapidly to 72 U/L. In trials performed using co-culture of *L. plantarum* A6 and *W. confusa* 17, the amylase production was evident after five h of fermentation. This mixed culture showed a maximum activity (3293 U/L) at ten h of fermentation. This enzymatic activity was higher than that reached by the mixed culture of *L. plantarum* A6 and *Sii*-25124 (2791 U/L). The α-amylase activity of *L. plantarum* A6 fermentation was the highest, while *W. confusa* 17 did not show activity. These results agree with the data obtained from the quantification of maltooligosaccharides, where an increase in starch hydrolysis products was not evident, as occurred with the cultures of *L. plantarum* A6 or *Sii*-25124 (Figure 4A–C).

### 3.4. Lactic Acid Production

The maximum lactic acid concentrations produced by *L. plantarum* A6, *Sii*-25124, and *W. confusa* 17 in pure culture were 17.90, 6.5, and 2 g/L, respectively. At 10 and 12 h of the assay, *L. plantarum* A6 in co-culture with *Sii*-25124 produced the highest lactic acid concentration (Figure 3B). However, in the co-cultures of *L. plantarum* A6 and *Sii*-25124 or *W. confusa* 17, the lactic acid concentration was lower than that reached as in pure culture, it was 17.90 g/L to 16 and 14 g/L, respectively (Figure 3B). On the contrary, in the co-culture of *Sii*-25124 and *W. confusa* 17, the lactic acid concentration increased to 8 g/L, compared to 6.5 g/L produced by *Sii*-25124 (Figure 3B).

### 3.5. Efficiency of Fermentation

As shown in Table 1, the highest lactic acid yield (Y_lac/s_) was produced by *L. plantarum* A6 (1.09 g/g), and the lowest Y_lac/s_ was obtained from the *W. confusa* 17 (0.32 g/g). Mixed cultures containing *L. plantarum* A6 reached Y_lac/s_ 0.8 g/g. In general, mono and co-cultures including *L. plantarum* A6 showed high values for biomass/substrate yield, α-amylase/biomass yield, α-amylase/substrate yield, and q_amy_ compared to *W. confusa* 17 and *Sii*-25124. In addition, the maximum specific growth rate (µ) was higher for *L. plantarum* A6 than *Sii*-25124 and *W. confusa* 17 (Table 1). However, when *W. confusa* 17 and *Sii*-25124 were mixed, the µ value increased 18% (Table 1), compared to *Sii*-25124 as pure cultures.

L.p, *Lactobacillus plantarum* A6 (new name *Lactiplantibacillus plantarum*); S.i, *S. infantarius* 25124; W.c, *Weisella confusa*. Biomass yield (Y_x/s_) was calculated as grams of biomass produced per gram of utilized sugar; lactic acid yield (Y_lac/s_) was calculated as grams of lactic acid produced per gram of utilized sugar; lactic acid yield (Y_lac/x_) was calculated as grams of lactic acid produced per gram of dry cell weight; amylase yield (Y_amy/x_) was calculated as units of amylase produced per gram of dry cell weight; amylase yield (Y_amy/s_) was calculated as units of amylase produced per gram of utilized sugar; µ: maximum specific growth rate; q_lac_, specific rate of lactic acid production; q_s_, specific rate of substrate consumption; q_amy_, specific rate of amylase production. a, g of lactic acid/g of cell dry wt*h; b, g of substrate/g of cell dry wt*h; c, U/g of cell dry wt*h. nd, non-determined. These values are the mean ± standard deviation of three experiments. Different lowercase letters in the same column show significant differences according to the analysis of variance at *p* ≤ 0.05 (LSD test). 

## 4. Discussion

In pozol fermentation, starch from nixtamal has been reported to be the main carbon and energy source available to allow the development of microbiota [8,10]. Both amylolytic and non-amylolytic lactic acid bacteria have been identified [8,10]. It has been reported that after six h pozol fermentation, the concentration of non-amylolytic LAB is higher than that of ALAB [8] then ALAB should allow the growth of non-amylolytic LAB, as a result of starch degradation and maltodextrin and maltose production [10] which could act as substrate form non-LAB. In the present study, the effect of these bacteria in pure and mixed cultures on fermentation efficiency, α-amylase activity, dynamics, and microbial growth of pure and mixed cultures of amylolytic LAB and non-amylolytic LAB on MRS-starch broth were studied. MRS-starch broth was used due to the easiness of handling this as a model of starch fermentation instead of pozol nixtamal dough.

Results showed that the amylolytic strain *L. plantarum* A6 produced more biomass than *Sii*-25124 and *W. confusa*. *L. plantarum* A6 is known as a highly amylolytic strain that has been used in different processes to produce pearl milled soybean fermented gruels [23], pearl milled-porridge [24], and sorghum porridge [25]. In this work, *L. plantarum* A6 showed higher microbial growth and α-amylase activity than *Sii*-25124 and *W. confusa*. It is probably due to extracellular alpha-amylase and neopullulanase activities [24,26] and the presence of maltodextrin transporters in *L. plantarum* [26]. Also, amylopectin phosphorylase and alpha-glucosidase enzymes that could be involved in starch hydrolysis [24]. This is possibly why the mixed cultures that included *L. plantarum* A6 showed higher values of kinetic parameters of starch fermentation (Table 1), α-amylase activity, and microbial biomass (Figure 1 and Figure 3D) than the rest of the cultures. On the other hand, *Sii*-25124 growth was lower than that of *L. plantarum* A6. Therefore, it could be associated with the culture medium composition and bacteria adaptation. *Sii*-25124 also is adapted to metabolize xylan and arabinoxylan, the second-largest fermentable carbohydrate in nixtamal dough, contributing greatly to their predominance in pozol [27]. However, its initial growth rate was the highest. It has been reported that *Streptococcus* produces a high protein concentration during the first 9 h of pozol fermentation including enzymes for starch hydrolysis [28]. This is an important trait, as the antimicrobial activity would be present earlier than that of the other strains, it could result in better food safety quality, eliminating pathogens earlier. In the same way, *W. confusa* 17, being non-amylolytic, did not grow as a pure culture, but neither in the mixed cultures with the two amylolytic strains. Indicating that it does not depend entirely on the amylolytic activity of the ALABs. It has been reported that *W. confusa* can grow using xylan and xylooligosaccharides as a carbon source [29], but also, it has the genes needed to metabolize sucrose, cellulose, hemicellulose, and starch residues, common carbohydrates in nixtamal dough [16]. This could be the reason for its predominance in pozol dough. However, the media used in this work did not contain any of these carbohydrates.

*W. confusa* 17 did not present growth nor amylolytic activity. However, the presence of this LAB in mixed culture with *Sii*-25124 resulted in the increase of Y_lac/s_ and q_amy_ values (Table 1) compared to A6 pure culture. This observation suggests that there is an interaction between both microbial populations when they coexist. Thus, lactic acid production from the mixed culture of these pozol LAB was higher than the one reached as monoculture. Humblot et al. [24] suggested that the expression of amylase genes varies depending on the starchy matrix. So, the enzymatic activity of these pozol strains could be higher in the dough than in MRS-starch broth. In this case, starch was not consumed completely, leaving approximately 30% of residual starch concentration, similarly to its concentration in pozol [10].

The amylolytic activity of *L. plantarum* A6 was higher than that of pozol LAB, possibly due to the higher biomass production in the medium. However, when this strain was inoculated in mixed culture with pozol LAB, its amylolytic activity, values of Y_lac/s_ (from 1.09 g/g to 0.82 g/g), q_amy_ (from 577.37 to 177.89), and µ (from 0.43 h^−1^ to 0.11 h^−1^) decreased (Table 1). Diaz-Ruíz et al. [8] suggested that the predominance of the non-amylolytic LAB in pozol fermentation was due to the amylolytic activity of ALAB, which releases sugars. The results obtained in this study showed that *Weissella confusa* 17 grew neither in MRS-starch broth nor in the same medium in co-culture with each of the amylolytic strains, and this suggests it does not depend on the sugars released by these bacteria. As *W. confusa* 17 does not grow in the medium or becomes non-culturable. Instead, it depends, most likely, on other carbohydrates present in pozol dough, such as those reported by Cooper-Bribiesca et al. [27] and López-Hernández et al. [29]. Both authors have found that *Sii*-25124 and *W. confusa* 17 can metabolize polysaccharides derived from hemicellulose, a carbohydrate present in the maize pericarp, and in nixtamal.

Single cultures kinetic data of *Sii*-25124, *L. plantarum* A6 y *W. confusa* 17 were different than those obtained with their co-cultures. Results indicate interactions among them. However, it is still unknown what kind these interactions are.

In combination with *L. plantarum* A6, both *Sii*-25124 and *W. confusa* 17 had a negative influence on the amylolytic activity, lactic acid yield (Y_lac/s_), specific growth rate (µ), and specific rate of amylase production (q_amy_) in the fermentation. Nevertheless, RT-PCR analysis showed that when *W. confusa* 17 was co-inoculated with the other two ALAB, it does not significantly increase its growth. These results suggest that the growth of the non-ALAB in pozol does not entirely depend on the amylase activity of the ALABs. It could be associated with the protein from maize (α-amylase and β-glucosidase), which release simple sugars for growth of microorganisms in the early stage of the pozol fermentation [28].

## 5. Conclusions

Clear differences in growth and fermentation parameters were found among pure and mixed cultures of the three lactic acid bacteria. *L. plantarum* A6 dominated in all cases, and *W. confusa* 17 did not grow. *Sii*-25124 dominated in co-culture with *W. confusa* 17 in MRS-starch broth. *L. plantarum* A6 fermentation parameters were higher than those of pozol LAB. However, growth rate of *Sii*-25124 in the first hours of fermentation was higher than that of the other bacteria. The advantages of growing in mixed cultures were: In *L. plantarum* A6-*Sii*-25124 mixed culture fermentation, the initial growth rate was higher than *L. plantarum* in pure culture, although biomass was lower. While *W. confusa* 17 affected the growth of *L. plantarum* A6, as more biomass was produced in its presence. Then, there are interactions among the strains studied, and the nature of these need to be investigated. Starch is essential for nixtamal dough fermentation, but it is possible that the growth of microorganisms also depends on other carbon sources present in dough.

This work contributes to the understanding of the interactions of amylolytic and non-amylolytic LAB in pozol.

## Figures and Tables

**Table 1:** Kinetic parameters of starch fermentation by pure and mixed culture of LAB. Different lowercase letters in the same column show significant differences according to the analysis of variance at *p* ≤ 0.05 (LSD test).

Inoculum	Parameters								
	Y_x/s_	Y_lac/s_	Y_lac/x_	Y_amy/x_	Y_amy/s_	µ	q_lac_^a^	q_s_^b^	q_amy_^c^
	g/g	g/g	g/g	U/g	U/g	h^−1^			
*L. plantarum*	0.24 ± 0.03 ^d^	1.09 ± 0.13 ^e^	4.57 ± 0.02 ^b^	1348.53 ± 20.40 ^c^	323.39 ± 40.82 ^bc^	0.43 ± 0.00 ^d^	1.95 ± 0.01 ^c^	1.79 ± 0.20 ^b^	577.37 ± 8.74 ^d^
*S. infantarius*	0.09 ± 0.00 ^b^	0.50 ± 0.00 ^b^	5.27 ± 0.03 ^c^	200.74 ± 3.15 ^b^	18.73 ± 0.50 ª	0.28 ± 0.02 ^b^	1.49 ± 0.07 ^b^	2.96 ± 0.04 ^d^	56.75 ± 0.89 ^a^
*W. confusa*	0.027 ± 0.00 ^a^	0.32 ± 0.02 ^a^	10.21 ± 0.05 ^f^	nd	nd	0.27 ± 0.01 ^b^	2.71 ± 0.13 ^e^	9.70 ± 0.16 ^e^	nd
*L.p-S.i*	0.22 ± 0.01 ^d^	0.82 ± 0.00 ^cd^	3.73 ± 0.16 ª	1668.66 ± 2.11 ^d^	334.21 ± 1.05 ^c^	0.11 ± 0.01 ª	0.4 ± 0.02 ª	0.48 ± 0.02 ^a^	177.89 ± 0.22 ^b^
*L.p-W.c*	0.15 ± 0.00 ^c^	0.86 ± 0.02 ^d^	5.91 ± 0.11 ^d^	1232.33 ± 173.22 c	280.71 ± 14.62 ^b^	0.33 ± 0.01 ^c^	1.94 ± 0.03 ^c^	2.26 ± 0.02 ^c^	404.00 ± 56.85 ^c^
*S.i-W.c*	0.11 ± 0.00 ^b^	0.72 ± 0.02 ^c^	7.43 ± 0.02 ^e^	303.85 ± 16.80 ^b^	27.16 ± 1.34 ^a^	0.33 ± 0.01 ^c^	2.47 ± 0.08 ^d^	2.90 ± 0.14 ^d^	101.03 ± 4.86 ^a^
